# Natural Molecule-Derived Nanogels from Hematoxylin and l-lysine for Biomedical Use with Antimicrobial Properties

**DOI:** 10.3390/ijms26010138

**Published:** 2024-12-27

**Authors:** Mehtap Sahiner, Zhi Tian, Diane Allen-Gipson, Aydin K. Sunol, Nurettin Sahiner

**Affiliations:** 1 Department of Bioengineering, Engineering Faculty, Canakkale Onsekiz Mart University, Terzioglu, Terzioglu Campus, 17100 Canakkale, Turkey; sahinerm78@gmail.com; 2 Department of Chemical and Biomolecular Engineering, University of South Florida, Tampa, FL 33620, USA; asunol@usf.edu; 3 Department of Pharmaceutical Sciences, College of Pharmacy, University of South Florida, Tampa, FL 33612, USA; ztian@usf.edu (Z.T.); dallengi@usf.edu (D.A.-G.); 4 Division of Allergy and Immunology, Department of Internal Medicine, College of Medicine, University of South Florida, Tampa, FL 33612, USA; 5 Department of Chemistry, Faculty of Sciences, Canakkale Onsekiz Mart University, Terzioglu Campus, 17100 Canakkale, Turkey; 6 Department of Ophthalmology, Morsani College of Medicine, University of South Florida, 12901 Bruce B. Downs Blvd, MDC21, Tampa, FL 33612, USA; 7 Department of Bioengineering, U.A. Whitaker College of Engineering, Florida Gulf Coast University, Fort Myers, FL 33965, USA

**Keywords:** natural molecules, nanoparticles, phenolic compounds, amino acid nanogels, biocompatible, biodegradable, l-lysine and hematoxylin, nanogels, microgels

## Abstract

Hematoxylin (HT) is a natural staining dye used in histopathology, often combined with Eosin for H&E staining. A poly(hematoxylin-co-l-lysine) (p(HT-co-l)) nanonetwork was synthesized through a one-step Mannich condensation reaction using formaldehyde as a linking agent. The resulting p(HT-co-l) nanogels had an average size of about 200 nm and exhibited a smooth surface and desirable functional groups such as -OH, -NH_2_, and -COOH, as recognized by FT-IR analysis. The isoelectric point (IEP) of the p(HT-co-l) nanogel was determined as pH 7.9, close to physiological environments, despite HT being acidic IEP at pH 1.7 and l-lysine being basic IPE at pH 8.7. The time-dependent swelling studies of p(HT-co-l) nanogels were carried out using dynamic light scattering (DLS) in different salt solutions, e.g., MgCl_2_, KNO_3_, KCl, PBS, and DI water environments revealed that nanogels have high swelling ability depending on the medium, e.g., >10-fold in a saline solution compared to distilled water within 1.5 h. Hydrolytic degradation studies in PBS demonstrated a linear release profile up to 125 h at 37.5 °C. The p(HT-co-l) nanogels also demonstrated significant antimicrobial and antifungal activities against *E. coli* (ATCC 8739), *S. aureus* (ATCC 6538), and *C. albicans* (ATCC 10231). Furthermore, biocompatibility tests indicated that p(HT-co-l) nanogels are more biocompatible than HT alone, as tested with human Nuli-1 bronchial epithelial cells.

## 1. Introduction

Hematoxylin (HT) is a wood-source flavonoid belonging to the polyphenol family comprising aromatic rings. HT is widely adapted for dyeing material in histology, cytology analyses, and histochemistry [1,2,3]. It has been studied for its electrochemical properties and applications in biosensors [3]. In a study, HT-modified carbon nanotubes were used to detect sulfide using the amphoteric method [4]. In general, HT is extensively utilized in diagnosis; however, investigations into its cure for treatment purposes are limited. Several reports have elucidated important pharmacological features of hematoxylin, including its antibacterial, anticoagulant, and anti-cancer effects [5]. In our prior study, we reported that HT is an antioxidant and inhibits alpha-glucosidase enzyme [6]. Interestingly, even metal-organic frameworks (MOFs) were prepared from Cu(II), Fe(II), and Fe(III) ions using HT as an organic linker [7]. Moreover, the blend of HT and Fe3+ ions is reported to exhibit notable anti-cancer properties when applied in photo-thermal therapy [8]. Studies have also explored HT interactions with metal ions and their photo-oxidation in oxygenated solutions as a bioactive flavonoid [9].

l-lysine amino acid is present in high-protein foods such as eggs, meat, soybeans, etc., as an ample and natural amino acid with a basic character that the human body cannot synthesize. Therefore, lysine is an essential fatty amino acid in humans and mammals. l-lysine has many metabolic functions, including the production of proteins and the regulation of immune responses [10]. l-lysine promotes muscle growth, boosts immunity, resists viruses, promotes fat oxidation, relieves anxiety, and improves nutrient absorption and performance [11]. It is one of the proteinogenic amino acids promoting muscle growth [12]. Moreover, l-lysine enhances pain sensitivity and effective defense after intraperitoneal and intracerebroventricular administration, influencing selective brain activity based on the biological significance of pain-induced behavior [13]. External food, particularly plant-based foods, is necessary for the absorption of l-lysine [14]. Humans get their l-lysine from the digestion of proteins that contain it, not from free l-lysine found in plant or animal cells. A lack of l-lysine in the body causes several health issues, including chronic malnutrition, anemia, fatigue, delayed growth, and problems with calcium absorption and antibody production [15]. l-lysine-based small molecules demonstrated broad-spectrum antibacterial activity as well as biofilm destruction [16]. Furthermore, the l-lysine molecule’s free amine and carboxyl groups can act as hydrogen bond donors and acceptors, making it simple to create hydrogen bonds with hydrophilic molecules, which increases its mechanical strength and adhesion [15,17]. Because of these natural characteristics, hydrogels based on l-lysine have been extensively researched in recent years [15].

Nanogels, as the smaller sizes of hydrogels, can afford high hydration capability and the ability to shrink or swell under various conditions faster. Their smaller 3D dimension, about 200 mm, allows them to encapsulate hydrophobic or hydrophilic drugs, protecting them from degradation during storage or in the body [18]. There are studies in the literature on the synthesis of nanoscale particles by dissolving various polyphenol structures in water, forming functional groups with formaldehyde, and then reacting with amino acids such as glycine and lysine [19,20]. This three-component, via the Mannich reaction, produces linear polyphenol oligomer derivatives that self-assemble into polyphenol nanoparticles [19]. There is a study on producing a stable nano-sized green sunscreen by adding the UV absorber aminobenzoic acid to the formaldehyde-modified form of EGCG using the same method [21]. When a lipid-soluble 9-fluorene-methoxycarbonyl motif was added to the polyphenol structure of epigallocatechin (EGCG), the resulting EGCG-Fmoc demonstrated a marked increase in lipid solubility and stability [22].

Here, p(HT-co-l) nanonetworks were generated by integrating HT with another benign biomolecule, l-lysine (l), via a Mannich condensation reaction, and self-assembling networks were prepared. p(HT-co-l) nanogels were examined for their swelling ability using Dynamic Light Scattering (DLS) in various salt environments, including CaCl_2_, MgCl_2_, KNO_3_, KCl, PBS, and DI water. Using the ECIS method, p(HT-co-l) nanogels were tested for biocompatibility against human Nuli-1 bronchial epithelial cells.

## 2. Results and Discussion

### 2.1. Characterization of p(HT-co-l) Nanogels

**Figure 1:**
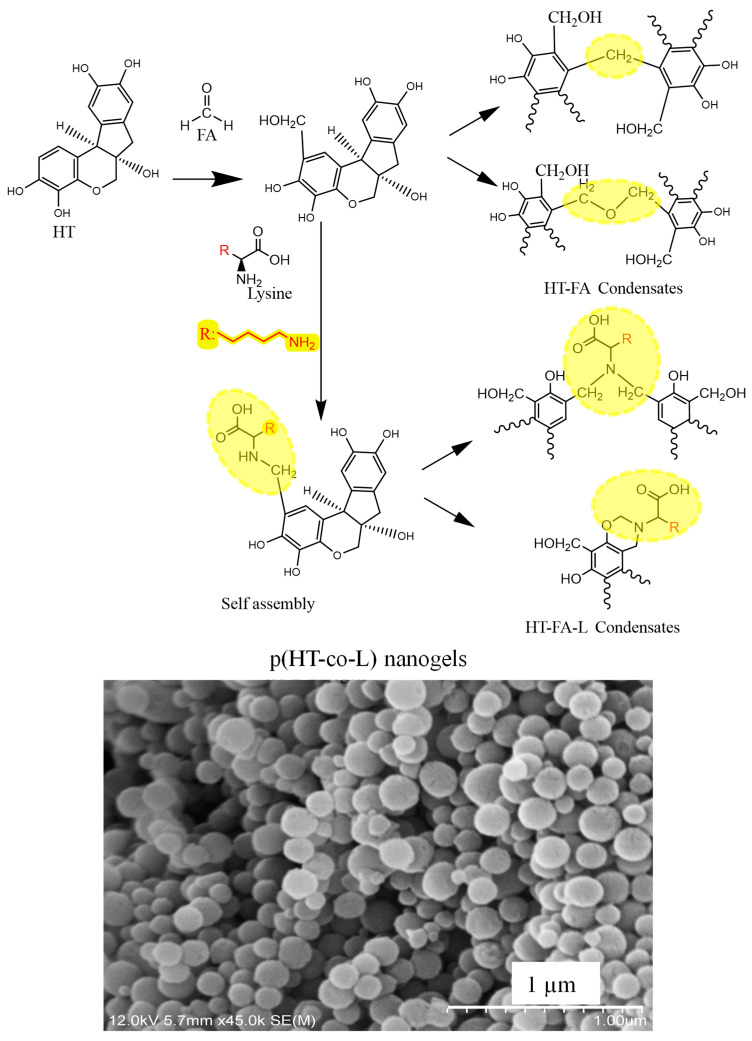
Illustration of the suggested mechanism for p(HT-co-l) nanogels formation and the corresponding SEM images of p(HT-co-l) nanogels.

The nanogel syntheses were performed using three different l-lysine ratios; only the reaction with 200 mol% l-lysine of HT was successful. In other words, the nanogels with other lysine ratios, e.g., 50% and 100% based on HT molecules, were not successful. The obtained p(HT-co-l) nanogels were spherical with smooth surfaces, as shown in Figure 1 with the corresponding SEM images. The literature reports similar reactions of resorcinol, a phenolic structure, with formaldehyde (FA) under acidic and basic conditions [23]. It has been mentioned that methylene bridges are formed in acidic conditions, and in basic conditions, methylol resorcinols are formed [23]. HT, which has a similar structure, is acidic in our reaction and is expected to have the possibility of methylene bridges at the end of FA addition as the anticipated reaction mechanism presented in Figure 1.

p(HT-co-l) nanogels were synthesized by the reaction of HT with Lysine (L) via a Mannich condensation reaction, and self-assembling networks were generated. The synthesized nanogels have spherical and smooth surfaces. The average diameter of p(HT-co-l) nanogels was determined as 163 ± 33 nm with SEM images via the Image J software program (Image J. 1.8.0). The gravimetric yield of the p(HT-co-l) nanogel was 57.28 ± 8.5%.

The intermolecular hydrogen bonds and π–π stacking forces enhanced the intermolecular entanglement and interaction of the derivatives, ultimately leading to the self-assembly of these oligomers into polyphenol nanoparticles.

**Figure 2:**
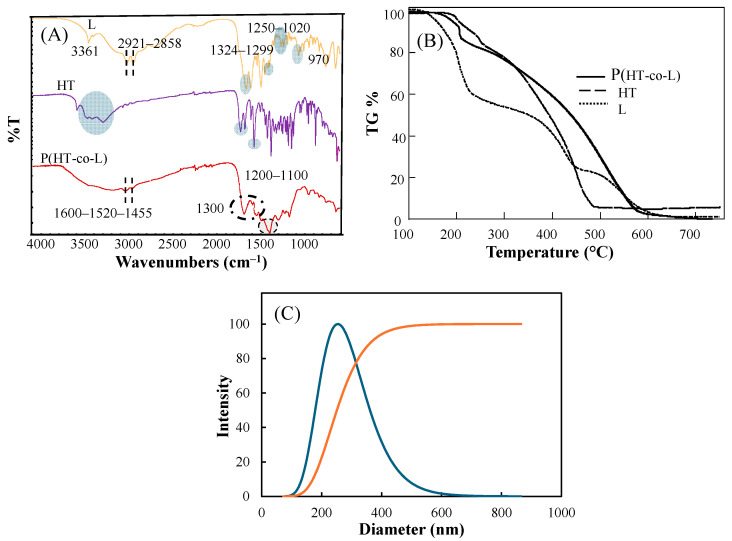
(**A**) FT-IR spectrum of l-lysine, HT, and p(HT-co-l) nanogels, (**B**) thermal degradation profile of L, HT, and p(HT-co-l) nanogels, and (**C**) size distribution of p(HT-co-l) nanogels in 10 mM KNO_3_ solution.

As shown in the FT-IR spectrum of the l-lysine (Figure 2A), the peak for –N–H stretching is at 3361 cm^−1^, and the aliphatic –CH_2_ peaks are at 2921 and 2858 cm^−1^. The –NH_2_ bending at 1567 cm^−1^, the C–O peaks at 1324 and 1299 cm^−1^, the C–N stretches at 1250–1020 cm^−1,^ and the OH bending of the carboxylic acid at 970 cm^−1^ are consistent with the literature.

The characteristic FT-IR peaks of HT were observed as –OH and –NH stretching vibrations at 3300–3600 cm^−1^, –C= C–vibrations at 1637 cm^−1^, –C–C–aromatic stretching vibrations at 1581 and 1513 cm^−1^, the –OH and –C–O deformation vibrations of phenolic e.g., –OH between 1335 and 1481 cm^−1^, –CO/–CH_2_/C–C vibrations between 1089 and 1290 cm^−1^, benzene ring stretching at 1038 cm^−1^, respectively and –C–H stretching of the benzene ring between 650 and 960 cm^−1^ are visible.

In the FT-IR spectrum of p(HT-co-l) nanogels, the aliphatic –CH_2_ peaks originating from L are maintained at 2921 and 2858 cm^−1^. The characteristic peaks at 1600, 1520, and 1455 cm^−1^ belong to the aromatic phenyl ring (C = C/C–C) of HT molecules. The peaks at 1360 cm^−1^ belong to the deformation vibration of C–OH bonds.

Thermal Gravimetric analysis given in Figure 2B for p(HT-co-l) nanogels, carried out up to 750 °C from 100 °C. TGA analysis revealed that 6.3wt% of the nanogels degraded at 200 °C, and 13.2 wt% degraded at 212 °C. At 601 °C, a total amount of 98.5wt% degraded. Approximately 20 wt% of lysine molecules degraded to 200 °C, but nearly half degraded at 374 °C. It was almost completely degraded at 750 °C with 1.2 wt% remaining. When heated to 251 °C, 14wt% of the HT molecule is degraded, while at 750 °C, the amount of degraded HT was 94.3 wt%.

As illustrated in Figure 2C, DLS measurement in 10 mM KNO_3_ revealed that the hydrodynamic diameter of p(HT-co-l) nanogels is 260 ± 12 nm in the swollen state with relatively homogenous size distributions in accordance with SEM images given in Figure 1. 

The elemental analyses of HT, L, and p(HT-co-l) nanogel were carried out to prove the formation of nanogel containing HT and L and the results are summarized in Table 1. The theoretical calculations and the elemental analysis results of C, H, N, and O% of p(HT-co-l) were summarized in Table 2. It was seen that all theoretically calculated and elemental analysis results for the determined C, H, N, and O elements of HT and L molecules are very similar except for O atoms. The amount of O in the experimental results is higher than the theoretical one. This may be due to the O in the air during the analysis. While the calculated C amount in p(HT-co-l) nanogels is 56.5%, the measured C amount is 54.1%, showing a yield of >95% by theoretical and experimental results.

**Figure 3:**
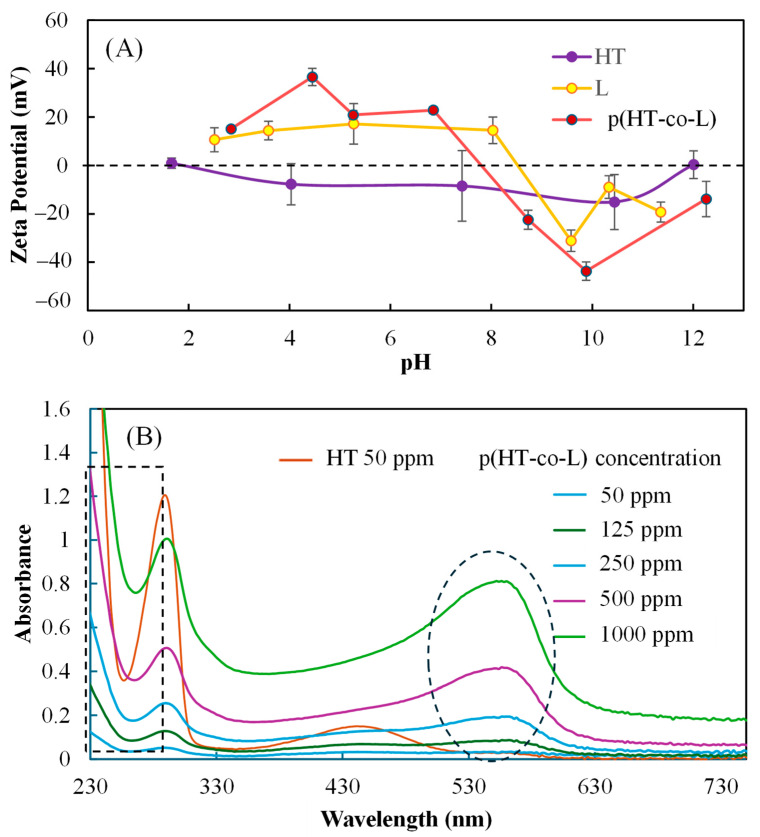
(**A**) Zeta potential versus solution pH values of p(HT-co-l), lysine, and HT and (**B**) the comparison of the UV-Vis spectrum of HT and p(HT-co-l) in deionized water at 50 and 1000 ppm concentration range.

In Figure 3A, the zeta potential values of HT, L, and P(HT-co-l) nanogels at different solution pHs were determined and compared. The IEP of HT, L, and p(HT-co-l) nanogels was determined from the graph. The IEP is the pH at which molecules or particles have net zero surface charges. The IEP of HT, l-L, and p(HT-co-l) nanogels was assessed at pH values of 1.8, 8.7, and 7.9, respectively. HT is a flavonoid that has an acidic character and has no IEP at above pH > 2. L, on the other hand, has a basic character with IEP at pH > 7. According to the report, L has an IEP of pH 9.47, is positively charged [24], and the reported value is close to our result. The synthesized p(HT-co-l) was found to be positively charged, about +20 mV at a physiological pH value of 7.4.

As shown in Figure 3B, a sharp UV peak (UV-Vis spectrophotometer) was observed for HT at 290 nm with an absorbance value of 1.2 for 50 ppm HT solution. For p(HT-co-l) nanogels at the same wavelength, this peak was found to be significantly smaller. The absorbance values of 1000–50 ppm dispersed solutions of p(HT-co-l) nanogels were determined as 1.000, 0.506, 0.255, 0.128, and 0.051, respectively. Also, a wide broad peak appeared at a wavelength of 564 nm for p(HT-co-l) nanogels. The absorption values at a concentration of 1000–50 ppm of the dispersed solutions of p(HT-co-l) nanogels were determined to be 0.797, 0.407, 0.191, 0.128, and 0.085, respectively, from UV-visible spectra at 564 wavelengths. These UV peaks of the nanogels are similar to the wavelengths of some blue pigments [25]. HT has a very low absorbance of 0.028 at 564 nm wavelengths.

**Figure 4:**
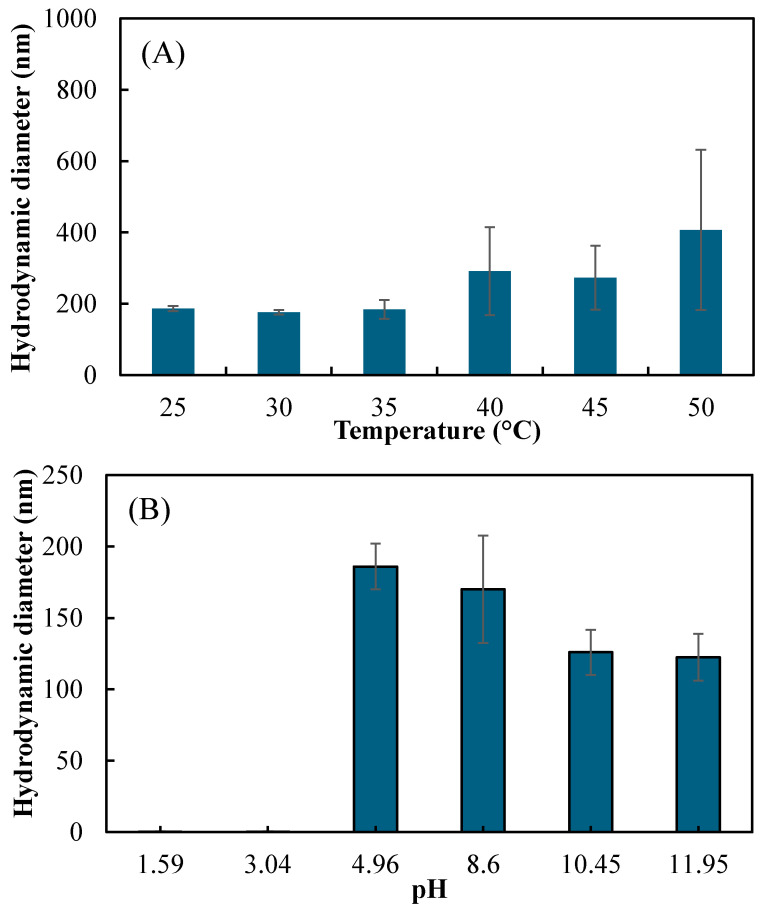
The change in size of p(HT-co-l) nanogels (**A**) at different temperatures and (**B**) at different pHs via DLS measurements.

In Figure 4A, the size of p(HT-co-l) nanogels does not change between 25 and 40 °C. After 40 °C, the size appears to increase in DLS. This may be due to the aggregation of nanogels. Therefore, p(HT-co-l) nanogels are not thermo-responsive. Figure 4B shows the size analysis at different pH values. In the range from pH 5 to 12, the hydrodynamic diameter of p(HT-co-l) nanogels decreases almost linearly. The sizes of p(HT-co-l) nanogels at pH 4.96 was 186 ± 5 nm, and at pH 12 it was 122 ± 11 nm. This is reasonable as Lysine units are deprotonated at a higher pH solution; the particle size gets smaller. At low pH, on the other hand, e.g., at pH 1.5–3, L units are protonated and swell extremely rupturing nanonetwork that decomposes the nanogels. Also, p(HT-co-l) nanogels give different colors, from yellow to purple, in different pH solutions. This color change is clearly visible, as given in Supplementary Figure S1.

**Figure 5:**
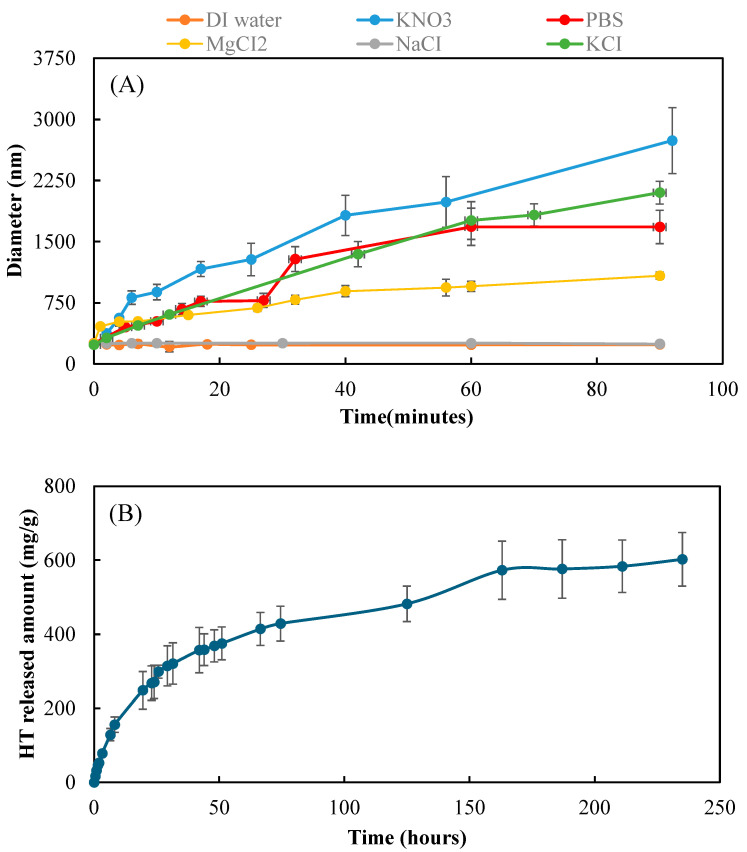
(**A**) The swelling of p(HT-co-l) nanogel in 10 mM different salt solutions for up to 90 min and (**B**) HT release in PBS solution at 37.5 °C.

Dynamic light scattering (DLS) was used to measure the size change of p(HT-co-l) nanogel swellings in 10 mM KNO_3_, MgCl_2_, KNO_3_, KCl, PBS, and DI water solutions as demonstrated in Figure 5A.

Nanogels do not swell in DI water, and 10 mM NaCl with time and dimensions are approximately 243 ± 6 nm. The swelling of p(HT-co-l) nanogels swelling time in some salt environments at 10 mM concentrations was measured and presented in Figure 4A. The fastest swelling occurred in the KNO_3_ solution, followed by KCl, PBS, and MgCl_2_. After 1.5 h in a KNO_3_ solution, the p(HT-co-l) nanogels size was measured as 2738 ± 404 nm, and 1 in PBS, the nanogels’ size was measured as 1629 ± 204 nm.

HT release from p(HT-co-l) nanogels at 37.5 °C was investigated in a PBS environment. Figure 5B shows the HT release was almost linear up to 75 h, and the cumulative release at 163 h was determined as 587 ± 78 mg HT/g nanogel. So, the p(HT-co-l) nanogels are very useful biomaterials for cell staining and long-term antioxidant applications in many biomedical uses.

### 2.2. Bioactive Properties of p(HT-co-l) Nanogels

According to the literature, HT and lysine derivates are natural structures with antibacterial properties [7,26]. The antimicrobial activity of lysine and p(HT-co-l) nanogels against two bacteria and one fungus was studied at a 10–0.6125 mg/mL concentration range. As shown in Table 2, L has a MIC against *E. coli* and *S. aureus* bacteria at a 10 mg/mL concentration. p(HT-co-l) nanogels have a MIC of 1.25 mg/mL and an MBC value of 5 mg/mL against *E. coli*. Similar particles reported by employing catechin (CAT) and l-Lysine (LYS) using formaldehyde (FA) via a single-step Mannich condensation reaction to generate CAT-LYS networks in the 700–800 nm range did not provide antibacterial results [27].

**Table 2:** MIC and MBC values of L and p(HT-co-l) nanogels against *E. coli* (ATCC 8739) [28], *S. aureus* (ATCC 6538) [29], and *C. albicans* (ATCC 10231) [30].

	*E. coli*		*S. aureus*		*C. albicans*	
	L	p(HT-co-l)	L	p(HT-co-l)	L	p(HT-co-l)
MIC	10	1.25	10	1.25	5	5
MBC	N.D	5	N.D	10	N.D	10

The MIC value of p(HT-co-l) nanogels against *S. aureus* was determined as 1.25 mg/mL, and the MBC value was 10 mg/mL. The MIC value of L was 10 mg/mL. In the test against the fungus *C. albicans* using the microdilution method, the MIC value of L was determined as 10 mg/mL. The MIC value of p(HT-co-l) against this yeast is also 5 mg/mL, and the MBC value is 10 mg/mL. So, it is obvious that p(HT-co-l) nanogels show better antibacterial properties than the used materials to make them, L and HT. This study affirms the innately antibacterial material characteristics of the prepared p(HT-co-l) nanogels that deliver high biomedical significance in using these materials as drug delivery systems, including delivery of antifungal and antibiotics for the treatment of various diseases.

### 2.3. Cell Toxicity Tests

*(a)*  *Cell cytotoxicity test of p(HT-co-l) nanogels*. Nuli-1 cells were cultured in normal medium for 20 h, and then 62.5 μg/mL, 125 μg/mL, 250 μg/mL, 500 μg/mL, and 1000 μg/mL p(HT-co-l) were added to each well, respectively.
*(b)*  *Cell cytotoxicity assay of HT*. Nuli-1 cells were cultured in a normal medium for 20 h, and then 62.5 μg/mL, 125 μg/mL, 250 μg/mL, and 500 μg/mL HT were added to each well, respectively. The signals were recorded at 4000 Hz. Each condition was performed in triplicate.
*(c)*  Cell cytotoxicity assay of p(HT-co-l) and HT. Nuli-1 cells were cultured in a normal medium for 20 h, and then 125 μg/mL p(HT-co-l) or HT was added to each well. The signals were recorded at 4000 Hz. Each condition was performed in triplicate.
*(d)*  Evaluation of Nuli-1 cells response to p(HT-co-l) nanogels.

The ECIS system was used to determine whether the toxicity of p(HT-co-l) based nanogels could be dynamically quantified by observing in vitro the ability of p(HT-co-l) nanogels to disrupt established nuli-1 epithelial cell monolayers cultured on the ECIS microelectrodes. Cell viability % was calculated using the media as the control without the samples [31].

**Figure 6:**
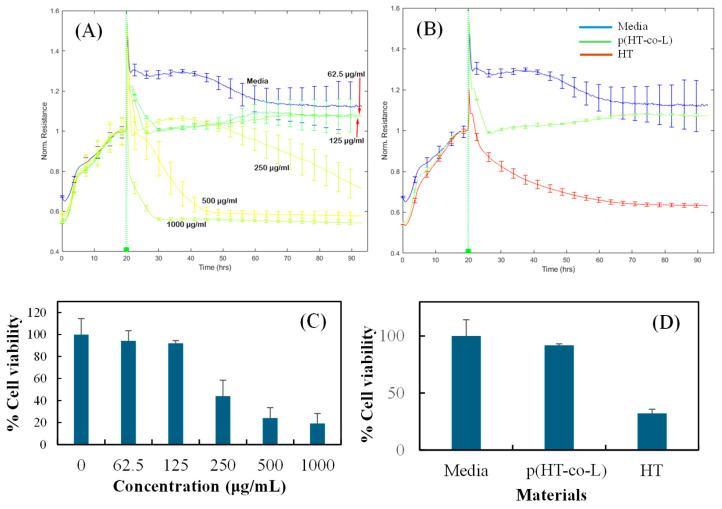
(**A**) p(HT-co-l) nanogels for 20 h and then 62.5 µg/mL, 125 µg/mL, 250 µg/mL, 500 µg/mL, and 1000 µg/mL of p(HT-co-l), (**B**) Cell cytotoxicity assay of p(HT-co-l) and HT for 20 h at the concentration of 125 µg/mL of HT-L or HT were added to each well, respectively. (**C**) The cell viability of Nuli-1 cells treated with various amounts of p(HT-co-l) nanogels for 72 h, and (**D**) The Cell viability of Nuli-1 cells treated with media, p(HT-co-l), and HT molecules at 125 µg/mL concentration for 72 h.

Cells contacted with p(HT-co-l) nanogel for 24, 48, and 72 h at lower concentrations ranging from 62.5–250 μg/mL revealed comparable resistance as compared to the media control, as shown in Figure 6A, but unfortunately, higher concentrations of p(HT-co-l) nanogel (500–1000 μg/mL) significantly reduced resistance as compared to media and reflectively demonstrated toxicity showed a significant reduction in cell viability as seen Figure 6A. The chemical structure of l-lysine (L) includes two amino groups and a carboxylic group. These groups can serve as drug-binding sites when functionalized onto nanoparticles [32,33]. HT only demonstrated reduced resistance, as previously observed [26,31]. However, adding l-lysine improved efficacy and increased resistance compared to media control, as presented in Figure 6B. Adding L also improved cell viability to 92% compared to HT, which has only 32% cell viability and was comparable to media control, as illustrated in Figure 5B. On the other hand, Figure 6C shows the cell viability of Nuli 1 cells after 72 h of contact in response to varying concentrations of p(HT-co-l) nanogels. The cell viabilities of 94.3 + 9.2 and 91.9 + 2.5% were obtained at p(HT-co-l) nanogels concentrations of 62.5 μg/mL and 125 μg/mL, respectively. Cell viability at a concentration of 250 μg/mL of nanogel was calculated as 44.1 + 14.3%. Figure 6D shows cell viability for media, p(HT-co-l) nanogel, and HT at a 125 μg/mL concentration at the end of 72 h. The p(HT-co-l) nanogel has a %cell viability of 91.9 + 2.5%, whereas the cell viability% of HT at the same concentration was 44.1 + 14.3%. l-lysine reduces HT toxicity and improves morphological changes without disrupting Nuli-1 monolayers. HT alone on Nuli-1 cells demonstrated concentration-dependent toxicity exposure at 72 h, revealing a significant reduction in cell viability for all concentrations ranging from 33% to 20%. The data obtained provided the importance of functionalizing nanoparticles to ensure bioavailability, efficacy, and safety in biological systems for potential biomedical use. The inclusion of L further increased HT safety and may prove to be an ideal gene/nucleic acid carrier.

Antibiotic resistance in *S. aureus* develops rapidly, and the emergence of multidrug-resistant strains is a major concern. Every year, over 10 million deaths are caused by antibiotic-resistant diseases, and by 2050, they are expected to surpass those caused by cancers. As traditional antibiotics are losing effectiveness due to bacterial resistance, there is an urgent need for new and effective solutions to reduce morbidity and mortality for pathogen-caused diseases. Therefore, alternative treatments are being investigated as a promising field due to the lack of new antibiotic classes [34]. Various strategies have been employed to inhibit virulence factors, including drug design with synthetic analogs, targeted delivery, and controlled release systems. Still, these studies have not yet produced promising results due to toxicity and/or low bioavailability [35]. Nanogels with tunable functionality derived from natural sources such as HT and L offer a promising approach to enhance the solubility, penetrability, stability, pharmacokinetic development, and overall therapeutic effectiveness of bioactive compounds. The specific size, e.g., below 200 nm, and shape range of the nanoparticulate delivery vehicles allow for significant targeting ability in the body [36]. In this study, natural, non-toxic, and antimicrobial nanosized, monodisperse, and spherical structures derived from natural plant-based material, HT, and an essential amino acid (lysine) were prepared and used as versatile biomaterials.

## 3. Materials and Methods

### 3.1. Materials

Hematoxylin Crystalline (HT, Fisher chemical, 85%, Geel, Belgium), (S)-2,6-Diaminohexanoic acid (l-lysine, Ambeed, 98%, Arlington, IL, USA) formaldehyde (FA, 37% by weight, With Preservative/Certified ACS, Fisher Chemical™, Geel, Belgium), were used to synthesize p(HT-co-l) nanogels.

Folin–Ciocalteau’s phenol reagent (FC, Sigma-Aldrich, St. Louis, MO, USA), Sodium nitrite (Merck, extra pure), and aluminum chloride (Merck, Rahway, NJ, USA, anhydrous powder sublimed from synthesis), gallic acid (GA, 97.5–102.5%, Aldrich), rosmarinic acid (RA, 96%, Aldrich) were used for antioxidant assays.

The antibacterial activity of p(HT-co-l) nanogels was evaluated against E. coli (ATCC 8739), S. aureus (ATCC 6538), and C. albicans (ATCC 10231). These microorganisms were purchased from KWIK-STIK Microbiologics in St. Cloud, MN, USA. Nutrient broth (RPI, 95%<, powder, RPI, Mount Prospect, IL, USA), nutrient agar (NA, dehydrated solid, BD DifcoTM, Sparks, MD, USA), and potato dextrose agar (PA, dehydrated solid, BD DifcoTM, Sparks, MD, USA) are used as microorganism medium bases.

### 3.2. Synthesis and Characterization of p(HT-co-l) Nanogels

p(HT-co-l) nanogels were synthesized in DI water according to the literature with some modifications [19,27]. Firstly, 30 mg HT (~0.1 mmol) was dissolved in 25 mL water at 500 rpm. Then, formaldehyde at 100 mol% of HT was added and mixed for another 30 min. Then, l-lysine at 100%, 200%, and 400 mol% was added to this mixture. After a reaction time of 4 h at room temperature (22 °C), it was precipitated by centrifugation at 10,000 rpm. The obtained nanosized p(HT-co-l) nanogels were washed in DI water and precipitated at 10,000 rpm for 25 min. After washing with water twice, the obtained nanogels were analyzed with Dynamic Light Scattering (DLS, Brookhaven Nanobrook Omni, New York, NY, USA). for size analysis. The prepared p(HT-co-l) nanogels were heat fan-dried and stored in a closed environment for later use and characterization tests.

The p(HT-co-l) nanogels were imaged using scanning electron microscopy (SEM, Hitachi Ultra High-Resolution Analytical FE-SEM SU-70, Tokyo, Japan). SEM images were acquired at 6–12 kV by mounting p(HT-co-l) nanogels onto carbon tape-attached aluminum SEM stubs coated with gold and mounted in a vacuum.

Fourier Transform Infrared Irradiation spectroscopy (FT-IR, iS10, Thermo Scientific, Waltham, MA, USA) was used to identify the chemical functional groups on p(HT-co-l) nanogels. The FT-IR spectra were collected after four repeated scans on all powdered nanogels using the ATR technique. FT-IR analysis was studied in the range of 4000–600 at room temperature.

Thermogravimetric Analyzer (TGA, SII TG/DTA6300, Exstar, Ibaraki, Japan) was employed for thermogravimetric analysis of p(HT-co-l) nanogels. The thermograms were obtained from p(HT-co-l) nanogels at 4 mg weights in a TG pan by heating from 100 to 750 °C at a rate of 10 °C/min in a nitrogen environment.

For the determination of the UV peak of p(HT-co-l) nanogels, HT, and L, a UV–Vis spectrophotometer (Thermo Scientific Genesys 180) was used. Zeta potential measurements were performed using a zeta potential meter with 40 mg of HT, L, and p(HT-co-l) nanogels suspended in 40 mL of 1 mM KNO_3_ solution, also using DLS (Brookhaven, Nanobrook Omni, New York, USA). The size measurements of p(HT-co-l) nanogels were carried out using DLS in 10 mM KNO_3_, MgCl_2_, KNO_3_, KCl, PBS, and DI water solutions. The particles are measured on the DLS device on the same day they are synthesized after washing. The hydrodynamic analysis as sizes was performed on freshly prepared nanogels without drying.

The HT release via hydrolytic degradation of p(HT-co-l) nanogels was examined at pH 7.4 in PBS. In these experiments, p(HT-co-l) nanogel weighing 0.010 g was suspended in 1 mL of PBS solutions and transferred to a dialysis membrane (molecular weight cutoff 12,000 Da, Aldrich). The dialysis membrane was then placed in a closed beaker containing 30 mL of PBS solution in a shaking water bath at 37.5 °C at 80 rpm. The released amount of HT was determined using UV-Vis spectroscopy (T80 UV/Vis spectrometer, PG Ins. Ltd., Harare, Zimbabwe) by measuring the absorbance values of the elution solutions at 290 nm. The amounts of HT in the solution outside the dialysis tubing were determined from previously generated calibration curves at 290 nm at pH 7.4 for HT molecules. All experiments were performed in triplicate, and the results are reported as average values with standard deviation.

### 3.3. Antibacterial Studies of p(HT-co-l) Nanogels

In this study, the growth and survival effects of L and p(HT-co-l) nanogels on various microorganisms were investigated. Gram-positive *S. aureus* ATCC 6538, Gram-negative *E. Coli* ATCC 8739, and *Candida albicans* ATCC 10231 were used for micro-titer dilution by established protocols [37]. Following UV sterilization at 355 nm for 5 min, p(HT-co-l) nanogels and L were suspended in 1 milliliter of 0.9 percent NaCl solution, each weighing 20 milligrams. The samples containing nanogel were homogenized using sonication for 30 s before use. In nutrient broth (NB), microorganism suspensions were calibrated to 0.5 McFarland. In a 96-well plate, we filled each well with 0.1 mL of NB. In the first well of each column, we added 0.1 mL of p(HT-co-l) nanogels or L, and then we diluted it. Each well received 5 µL of bacteria suspension, which was then incubated for 24 h at 35 °C [6]. The MIC value was determined in nutrient broth. After the MIC value was determined, the samples were seeded in NB for 24 h of incubation time at 35 °C, then MBC values were determined.

### 3.4. Cell Culture

American Type Culture Collection (ATCC) cell line named Nuli-1 human bronchial epithelial cells were obtained from MarylandCells (Rockville, MD, USA) cultured on dishes precoated with placental collagen type VI (Sigma, St. Louis, MA, USA) in serum-free bronchial epithelial growth medium (BEGM; Lonza, Walkersville, MD, USA). Cells were maintained in a humidified incubator at 37 °C in a 5% CO_2_ atmosphere, as previously described. Cell images were captured using the EVOS XL M3000 imaging system (Life Technologies, Carlsbad, CA, USA).

### 3.5. Cell Cytotoxicity Assay

96W10idf ECIS culture vessels (Applied BioPhysics, Troy, NY) were used for cell cytotoxicity assay as previously described [38]. Electrical cell-substrate impedance measurement (ECIS, Applied BioPhysics Inc., Troy, NY, USA) is a non-invasive method for monitoring live animal cells in vitro. Cells are grown on small gold film electrodes placed in a Petri dish. Briefly, eight (103 cells) were seeded per well and cultured in an incubator at 37 °C in a 5% CO_2_ atmosphere. The media was changed the next day, and then, five different concentrations of HT, L, and p(HT-co-l) nanogels (62.5–1000 µg/mL) were added to each well. Resistances were recorded in real-time at 4000 Hz using an ECIS Zθ instrument (Applied BioPhysics, Troy, NY, USA).

## 4. Conclusions

p(HT-co-l) nanogels, synthesized through a well-established method, involved the integration of hematoxylin (HT) with lysine (L) via a Mannich condensation reaction. This chemical process facilitates the formation of self-assembling networks that organize effectively at the nanoscale, resulting in uniform nanogels with many potential in vivo applications, e.g., cell staining and drug-carrying ability as naturally antioxidant and antibacterial materials. The prepared p(HT-co-l) nanogels possess several advantages, including biocompatibility that secures their safe use in biological systems and biodegradability, allowing for nontoxic degradation product, HT and/or L, upon their in vivo applications. HT is an acidic natural phenolic compound and has an IEP of pH 1.7, whereas the amino acid L molecule is a basic amino acid with an IEP value of pH 8.7, and the IEP of the synthesized p(HT-co-l) nanogels was determined as pH 7.9, which is very close to the physiological pHs. Additionally, these nanogels exhibit significant intrinsic antibacterial properties, making them suitable for medical and pharmaceutical uses where microbial resistance is of paramount significance. Importantly, p(HT-co-l) nanogels are capable of releasing HT, a natural phenolic compound known for its potent antioxidant properties. This HT-releasing ability enhances not only the functionality of the nanogels but also positions p(HT-co-l) nanogels as valuable tools in therapeutic perspectives, particularly in drug delivery systems. As a result, p(HT-co-l) nanogels represent a multifunctional platform that holds viable platforms for advancing drug delivery technologies that address diverse therapeutic and complex needs in the medical field. Therefore, these unique combinations of properties of each component of p(HT-co-l) nanogels open new avenues for innovative treatments in both preventive and restorative healthcare applications. Keeping in mind that the components of p(HT-co-l), HT, and L are natural in origin, surmounting all environmental and biological concerns and providing the viable and sustainable scalability of these nanogels, they have promising future application potentials.

## Figures and Tables

**Table 1:** The elemental composition of synthesized p(hT-co-l) nanogels.

	Theoretical Calculation				Experimental			
	C%	H%	N%	O%	C%	H%	N%	O%
HT	63.3	4.6	0.0	32.1	54.8	4.9	0.0	40.0
L	49.3	9.6	19.1	22.0	46.3	7.5	15.4	30.8
p(HT-co-l)	56.5	7.1	4.7	31.7	54.1	5.9	6.3	33.7

## Data Availability

All the data created in this study remained within the study.

## Supplementary Materials

The following supporting information can be downloaded at https://www.mdpi.com/article/10.3390/ijms26010138/s1.

## References

[1] Drachneris J., Morkunas M., Fabijonavicius M., Cekauskas A., Jankevicius F., Laurinavicius A. (2024). Prediction of Non-Muscle Invasive Papillary Urothelial Carcinoma Relapse from Hematoxylin–Eosin Images Using Deep Multiple Instance Learning in Patients Treated with Bacille Calmette–Guérin Immunotherapy. Biomedicines.

[2] Shovon M.S.H., Islam M.J., Nabil M.N.A.K., Molla M.M., Jony A.I., Mridha M.F. (2022). Strategies for Enhancing the Multi-Stage Classification Performances of HER2 Breast Cancer from Hematoxylin and Eosin Images. Diagnostics.

[3] Cooksey C.J. (2021). Hematoxylin in the 21 St Century. Biotech. Histochem..

[4] Long Vu D., Červenka L. (2013). Determination of Sulfide by Hematoxylin Multiwalled Carbon Nanotubes Modified Carbon Paste Electrode. Electroanalysis.

[5] Yin H.-H., Han Y.-L., Yan X., Guan Y.-X. (2023). Hematoxylin Modulates Tau-RD Protein Fibrillization and Ameliorates Alzheimer’s Disease-like Symptoms in a Yeast Model. Int. J. Biol. Macromol..

[6] Sahiner M., Sunol A.K., Sahiner N. (2024). Cell Staining Microgels Derived from a Natural Phenolic Dye: Hematoxylin Has Intriguing Biomedical Potential. Pharmaceutics.

[7] Sahiner M., Tian Z., Demirci S., Sunol A., Allen-Gipson D.S., Sahiner N. (2024). Bio-MOFs Based on Natural Phenolic, Hematoxylin Leverages Biomedical Applications: Enzyme Inhibition, Antioxidant, and Antibacterial Properties. Chem. Biodivers..

[8] He X., Zhu H., Shang J., Li M., Zhang Y., Zhou S., Gong G., He Y., Blocki A., Guo J. (2022). Intratumoral Synthesis of Transformable Metal-Phenolic Nanoaggregates with Enhanced Tumor Penetration and Retention for Photothermal Immunotherapy. Theranostics.

[9] Zare H.R., Nasirizadeh N. (2011). A Study of the Electrochemical Behavior of Hematoxylin as an Important Bioactive Flavonoid. Electrochim. Acta.

[10] Wunderle C., Haller L., Laager R., Bernasconi L., Neyer P., Stumpf F., Tribolet P., Stanga Z., Mueller B., Schuetz P. (2024). The Association of the Essential Amino Acids Lysine, Methionine, and Threonine with Clinical Outcomes in Patients at Nutritional Risk: Secondary Analysis of a Randomized Clinical Trial. Nutrients.

[11] Tang S., Wei Z., Guo J., Sun X., Hu Y. (2023). Enantioselective Recognition of L-Lysine by ICT Effect with a Novel Binaphthyl-Based Complex. Micromachines.

[12] Thoma B., Powner M.W. (2023). Selective Synthesis of Lysine Peptides and the Prebiotically Plausible Synthesis of Catalytically Active Diaminopropionic Acid Peptide Nitriles in Water. J. Am. Chem. Soc..

[13] Severyanova L.A., Lazarenko V.A., Plotnikov D.V., Dolgintsev M.E., Kriukov A.A. (2019). L-Lysine as the Molecule Influencing Selective Brain Activity in Pain-Induced Behavior of Rats. Int. J. Mol. Sci..

[14] Zhao M., Lin Y., Chen H. (2020). Improving Nutritional Quality of Rice for Human Health. Theor. Appl. Genet..

[15] Gao Y., Jia F., Gao G. (2019). Transparent and Conductive Amino Acid-Tackified Hydrogels as Wearable Strain Sensors. Chem. Eng. J..

[16] Konai M.M., Haldar J. (2015). Lysine-Based Small Molecules That Disrupt Biofilms and Kill Both Actively Growing Planktonic and Nondividing Stationary Phase Bacteria. ACS Infect. Dis..

[17] Juan C.-Y., Zhang Y.-S., Cheng J.-K., Chen Y.-H., Lin H.-C., Yeh M.-Y. (2024). Lysine-Triggered Polymeric Hydrogels with Self-Adhesion, Stretchability, and Supportive Properties. Polymers.

[18] Yin Y., Hu B., Yuan X., Cai L., Gao H., Yang Q. (2020). Nanogel: A Versatile Nano-Delivery System for Biomedical Applications. Pharmaceutics.

[19] Yi Z., Chen G., Chen X., Ma X., Cui X., Sun Z., Su W., Li X. (2020). Preparation of Strong Antioxidative, Therapeutic Nanoparticles Based on Amino Acid-Induced Ultrafast Assembly of Tea Polyphenols. ACS Appl. Mater. Interfaces.

[20] Tian M., Chen G., Xu J., Lin Y., Yi Z., Chen X., Li X., Chen S. (2022). Epigallocatechin Gallate-Based Nanoparticles with Reactive Oxygen Species Scavenging Property for Effective Chronic Periodontitis Treatment. Chem. Eng. J..

[21] Chen X., Yi Z., Chen G., Ma X., Tong Q., Tang L., Li X. (2022). Engineered Fabrication of EGCG-UV Absorber Conjugated Nano-Assemblies for Antioxidative Sunscreens with Broad-Band Absorption. Colloids Surf. B Biointerfaces.

[22] Liu C., Wu H., Duan H., Hou Y., Wang S., Liu Y., Zhang X., Zhao H., Gong L., Wan H. (2023). An EGCG-Mediated Self-Assembled Micellar Complex Acts as a Bioactive Drug Carrier. Food Chem..

[23] ElKhatat A.M., Al-Muhtaseb S.A. (2011). Advances in Tailoring Resorcinol-Formaldehyde Organic and Carbon Gels. Adv. Mater..

[24] Ustunol I.B., Gonzalez-Pech N.I., Grassian V.H. (2019). PH-Dependent Adsorption of α-Amino Acids, Lysine, Glutamic Acid, Serine and Glycine, on TiO2 Nanoparticle Surfaces. J. Colloid Interface Sci..

[25] Singh A.V., Bansod G., Schumann A., Bierkandt F.S., Laux P., Nakhale S.V., Shelar A., Patil R., Luch A. (2024). Investigating Tattoo Pigments Composition with UV-Vis and FT-IR Spectroscopy Supported by Chemometric Modelling. Curr. Anal. Chem..

[26] Ye R., Xu H., Wan C., Peng S., Wang L., Xu H., Aguilar Z.P., Xiong Y., Zeng Z., Wei H. (2013). Antibacterial Activity and Mechanism of Action of ε-Poly-l-Lysine. Biochem. Biophys. Res. Commun..

[27] Can M., Sahiner M., Sahiner N. (2022). Colloidal Bioactive Nanospheres Prepared from Natural Biomolecules, Catechin and L-Lysine. J. Polym. Res..

[28] ATCC 8739. https://www.atcc.org/products/8739.

[29] ATCC 6538. https://www.atcc.org/products/6538.

[30] ATCC 10231. https://www.atcc.org/products/10231.

[31] Dash P., Nataraj N., Panda P.K., Tseng C.-L., Lin Y.-C., Sakthivel R., Chung R.-J. (2024). Construction of Methotrexate-Loaded Bi 2 S 3 Coated with Fe/Mn-Bimetallic Doped ZIF-8 Nanocomposites for Cancer Treatment Through the Synergistic Effects of Photothermal/Chemodynamic/Chemotherapy. ACS Appl. Mater. Interfaces.

[32] Badea I., Alwani S., Kaur R., Michel D., Chitanda J.M., Verrall R., Karunakaran C. (2016). Lysine-Functionalized Nanodiamonds as Gene Carriers: Development of Stable Colloidal Dispersion for in Vitro Cellular Uptake Studies and SiRNA Delivery Application. Int. J. Nanomed..

[33] Lukasheva E., Makletsova M., Lukashev A., Babayeva G., Arinbasarova A., Medentsev A. (2020). Fungal Enzyme L-Lysine α-Oxidase Affects the Amino Acid Metabolism in the Brain and Decreases the Polyamine Level. Pharmaceuticals.

[34] Joshi K.M., Shelar A., Kasabe U., Nikam L.K., Pawar R.A., Sangshetti J., Kale B.B., Singh A.V., Patil R., Chaskar M.G. (2022). Biofilm Inhibition in Candida Albicans with Biogenic Hierarchical Zinc-Oxide Nanoparticles. Biomater. Adv..

[35] Ahmad-Mansour N., Loubet P., Pouget C., Dunyach-Remy C., Sotto A., Lavigne J.-P., Molle V. (2021). Staphylococcus Aureus Toxins: An Update on Their Pathogenic Properties and Potential Treatments. Toxins.

[36] Taha M., Alhakamy N.A., Md S., Ahmad M.Z., Rizwanullah M., Fatima S., Ahmed N., Alyazedi F.M., Karim S., Ahmad J. (2022). Nanogels as Potential Delivery Vehicles in Improving the Therapeutic Efficacy of Phytopharmaceuticals. Polymers.

[37] Suner S.S., Sahiner M., Akcali A., Sahiner N. (2020). Functionalization of Halloysite Nanotubes with Polyethyleneimine and Various Ionic Liquid Forms with Antimicrobial Activity. J. Appl. Polym. Sci..

[38] Tian Z., Zhang H., Dixon J., Traphagen N., Wyatt T.A., Kharbanda K., Simet Chadwick S., Kolliputi N., Allen-Gipson D.S. (2017). Cigarette Smoke Impairs A2A Adenosine Receptor Mediated Wound Repair through Up-Regulation of Duox-1 Expression. Sci. Rep..

