# Supplementary material for: Natural Molecule-Derived Nanogels from Hematoxylin and l-lysine for Biomedical Use with Antimicrobial Properties
Source: Int J Mol Sci. 2024 Dec 27;26(1):138. doi: 10.3390/ijms26010138 (PMC11719568; doi:10.3390/ijms26010138)
Supplement: Supplementary file 1 [file ijms-26-00138-s001.zip › ijms-3366102-supplementary.pdf]

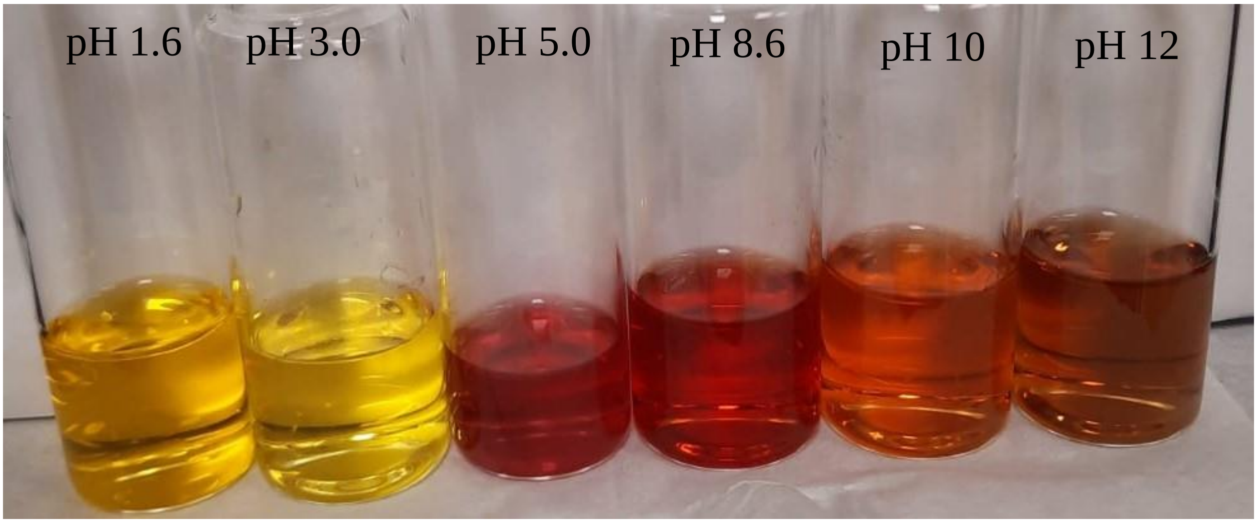

Supporting Figure S1. Suspension image of p(HT-co-L) nanogel in DI water at different pHs (pH adjusted with 0.1M NaOH and 0.1M HCl)
